# The state of health in Indonesia's provinces, 1990–2019: a systematic analysis for the Global Burden of Disease Study 2019

**DOI:** 10.1016/S2214-109X(22)00371-0

**Published:** 2022-10-11

**Authors:** GBD 2019 Indonesia Subnational Collaborators, Nafsiah Mboi, Ruri Syailendrawati, Samuel M Ostroff, Iqbal RF Elyazar, Scott D Glenn, Tety Rachmawati, Wahyu Pudji Nugraheni, Pungkas Bahjuri Ali, Laksono Trisnantoro, Qorinah Estiningtyas Sakilah Adnani, Rozana Ika Agustiya, Agung Dwi Laksono, Budi Aji, Luna Amalia, Ansariadi Ansariadi, Ernoiz Antriyandarti, Irfan Ardani, Ratih Ariningrum, Ni Ketut Aryastami, Djunaedi Djunaedi, Ferry Efendi, Nelsensius Klau Fauk, Ghozali Ghozali, Nariyah Handayani, Harapan Harapan, Arief Hargono, Agus DWI Harso, Hartanti Dian Ikawati, Endang Indriasih, Karlina Karlina, Soewarta Kosen, Asep Kusnali, Dian Kusuma, Melyana Lumbantoruan, Merry Lusiana, Santi Martini, Meilinda meilinda, Rofingatul Mubasyiroh, Dede Anwar Musadad, Dina Nur Anggraini Ningrum, Syarifah Nuraini, Ni'matun Nurlaela, Helena Ullyartha Pangaribuan, Indah Pawitaningtyas, Agung Purnomo, Herti Windya Puspasari, Nurul Puspasari, Setyaningrum Rahmawaty, Hasnani Rangkuti, Betty Roosihermiatie, Tita Rosita, Rustika Rustika, Yoseph Leonardo Samodra, Jenny Veronika Samosir, Siswanto Siswanto, Karen Houston Smith, Agus Sudaryanto, Sugianto Sugianto, Suparmi Suparmi, Ni Ketut Susilarini, Ingan Ukur Tarigan, Jansje Henny Vera Ticoalu, Indang Trihandini, Mugi Wahidin, Tati Suryati Warouw, Retno Widyastuti, Ratna DWI Wulandari, Simon I Hay, Christopher J L Murray, Ali H Mokdad

## Abstract

**Background:**

Analysing trends and levels of the burden of disease at the national level can mask inequalities in health-related progress in lower administrative units such as provinces and districts. We used results from the Global Burden of Diseases, Injuries, and Risk Factors Study (GBD) 2019 to analyse health patterns in Indonesia at the provincial level between 1990 and 2019. Long-term analyses of disease burden provide insights on Indonesia's advance to universal health coverage and its ability to meet the United Nations Sustainable Development Goals by 2030.

**Methods:**

We analysed GBD 2019 estimated cause-specific mortality, years of life lost (YLLs), years lived with disability (YLDs), disability-adjusted life-years (DALYs), life expectancy at birth, healthy life expectancy, and risk factors for 286 causes of death, 369 causes of non-fatal health loss, and 87 risk factors by year, age, and sex for Indonesia and its 34 provinces from 1990 to 2019. To generate estimates for Indonesia at the national level, we used 138 location-years of data to estimate Indonesia-specific demographic indicators, 317 location-years of data for Indonesia-specific causes of death, 689 location-years of data for Indonesia-specific non-fatal outcomes, 250 location-years of data for Indonesia-specific risk factors, and 1641 location-years of data for Indonesia-specific covariates. For subnational estimates, we used the following source counts: 138 location-years of data to estimate Indonesia-specific demographic indicators; 5848 location-years of data for Indonesia-specific causes of death; 1534 location-years of data for Indonesia-specific non-fatal outcomes; 650 location-years of data for Indonesia-specific risk factors; and 16 016 location-years of data for Indonesia-specific covariates. We generated our GBD 2019 estimates for Indonesia by including 1 915 207 total source metadata rows, and we used 821 total citations.

**Findings:**

Life expectancy for males across Indonesia increased from 62·5 years (95% uncertainty interval 61·3–63·7) to 69·4 years (67·2–71·6) between 1990 and 2019, a positive change of 6·9 years. For females during the same period, life expectancy increased from 65·7 years (64·5–66·8) to 73·5 years (71·6–75·6), an increase of 7·8 years. There were large disparities in health outcomes among provinces. In 2019, Bali had the highest life expectancy at birth for males (74·4 years, 70·90–77·9) and North Kalimantan had the highest life expectancy at birth for females (77·7 years, 74·7–81·2), whereas Papua had the lowest life expectancy at birth for males (64·5 years, 60·9–68·2) and North Maluku had the lowest life expectancy at birth for females (64·0 years, 60·7–67·3). The difference in life expectancy for males between the highest-ranked and lowest-ranked provinces was 9·9 years and the difference in life expectacy for females between the highest-ranked and lowest-ranked provinces was 13·7 years. Age-standardised death, YLL, and YLD rates also varied widely among the provinces in 2019. High systolic blood pressure, tobacco, dietary risks, high fasting plasma glucose, and high BMI were the five leading risks contributing to health loss measured as DALYs in 2019.

**Interpretation:**

Our findings highlight that Indonesia faces a double burden of communicable and non-communicable diseases that varies across provinces. From 1990 to 2019, Indonesia witnessed a decline in the infectious disease burden, although communicable diseases such as tuberculosis, diarrhoeal diseases, and lower respiratory infections have remained a main source of DALYs in Indonesia. During that same period, however, all-ages death and disability rates from non-communicable diseases and exposure to their risk factors accounted for larger shares of health loss. The differences in health outcomes between the highest-performing and lowest-performing provinces have also widened since 1990. Our findings support a comprehensive process to revisit current health policies, examine the root causes of variation in the burden of disease among provinces, and strengthen programmes and policies aimed at reducing disparities across the country.

**Funding:**

The Bill & Melinda Gates Foundation and the Government of Indonesia.

**Translation:**

For the Bahasa Indonesia translation of the abstract see Supplementary Materials section.

## Introduction

Indonesia is the fourth-most populous country in the world, with a population of about 260 million people.^1^ A multiethnic archipelago nation, Indonesia's island geography and vast population complicate efforts to solve health problems, strengthen health systems, achieve universal health coverage (UHC), and meet Sustainable Development Goal targets by 2030.^2^ In 1999, Indonesia started a decentralisation process that saw many governmental responsibilities, including health, devolved by the central government to provinces, districts, and municipalities. The latest transfer of power to local agencies came in 2018, when a government regulation vested provincial governors with greater authority for health and development programmes. Alongside these decentralisation efforts, Indonesia has implemented a series of health governance reforms. In 2005, the central government implemented the Social Health Insurance programme (Jaminan Kesehatan Sosial), which included an initiative to provide health insurance and services to low-income and underserved populations. A revamped version of the programme—the Community Health Insurance programme (Jaminan Kesehatan Masyarakat)—was launched 3 years later. In January, 2014, the central government established the National Health Insurance scheme (Jaminan Kesehatan Nasional) to provide insurance coverage to all Indonesians.^3^ Government programmes have also attempted to address disparities between provinces through a regional development initiative focused on the eastern provinces, which have been historically underserved and have poorer health outcomes. Despite these efforts, Jaminan Kesehatan Nasional has not met its goal of covering 95% of Indonesians by 2020, and earlier analyses suggest that Indonesian provinces remain unevenly developed despite such reforms.^4^

**Research in context**

**Evidence before this study**

The Global Burden of Diseases, Injuries, and Risk Factors Study (GBD) 2019 is a comprehensive analysis of health loss across the globe. Included in GBD 2019 is a quantification of health loss for Indonesia and its provinces from 1990 to 2019. The analysis contains estimates of deaths, years of life lost because of premature mortality, years of life lived with disability, and disability-adjusted life years attributable to metabolic, environmental, and occupational and behavioural risk factors at the national and subnational levels. National estimates were published on the basis of previous GBD releases. GBD estimates have been used by the Indonesian government, particularly the Ministry of Health, to examine national-level health performance and progress and to plan, develop, and implement programmes to improve health and eliminate disparities. No previous estimates of total health and health loss in Indonesia at the subnational level have been published.

**Added value of this study**

Presenting the burden of disease and its trends in Indonesia from 1990 to 2019 at the subnational level provides valuable information for stakeholders to improve health, shape policy, design interventions, and set funding priorities in pursuit of these goals. This is the first study to provide a comprehensive assessment of the burden of disease at the provincial level in Indonesia. Building on the success of previous GBD iterations being used by the Ministry of Health and other governmental agencies in Indonesia, a study of the burden of disease at the provincial level offers decision makers access to more resolved estimates to increase the specificity of policy effects. Since many health policy and management decisions are made at the district level in Indonesia, subnational estimates can better inform resource allocation and policy implementation. Indonesia started a decentralisation process in 1999. Responsibility for many fields of governance, including health, was devolved by the central government to provinces, districts, and municipalities. The latest transfer of power to local authorities came in 2018, when a government regulation vested provincial governors with greater responsibility for health and development programmes. Provincial officials will require reliable health data to make evidence-based decisions about policies and resource allocations that will reduce burden and help the country meet its universal health coverage (UHC) and Sustainable Development Goal (SDG) targets. Moreover, as the COVID-19 pandemic continues, the value of this GBD benchmarking is enhanced because it provides prepandemic historical context of health at the provincial level in Indonesia.

**Implications of all the available evidence**

The burden of disease profiles of Indonesia's 34 provinces present complex, granular pictures of subnational health of the fourth-most populous country in the world. Provincial health estimates provide valuable information to tackle the challenges of health policy and governance in the context of a global pandemic. Authority over health and development programmes has been devolved gradually by the central government to provincial and local administrations, which increases the need for estimates of health loss at the subnational level. Providing estimates of the burden of disease at the provincial level enables health professionals, policy makers, and stakeholders to address the leading causes of diseases, injuries, and deaths and to monitor progress on the country's path to UHC and meeting its SDG benchmarks.

The Global Burden of Diseases, Injuries, and Risk Factors Study (GBD) 2019 provides a comprehensive overview of health conditions in Indonesia immediately before the outbreak of SARS-CoV-2. Estimates from before COVID-19 create important baselines of disease burden in Indonesia at the national and subnational levels. Setting prepandemic baselines sheds light on the efficacy of policies implemented in Indonesia since 1990 that might otherwise go undetected because of the COVID-19 pandemic. At the same time, GBD 2019 provides insights on the uneven development across Indonesian provinces, and the varied successes of governmental programmes and schemes to improve health outcomes throughout 2019. Insights from this and previous GBD studies illuminate the double burden of communicable and non-communicable diseases that created the perfect conditions for COVID-19 in Indonesia.^5^ Many non-communicable diseases, such as diabetes, hypertension, and asthma, and their associated risk factors are comorbidities for COVID-19; excess mortality caused by COVID-19 has also been vastly underestimated during the pandemic.^6^ The persistence of eradicable infectious diseases in Indonesia, including malaria and tuberculosis, places further stress on the country's health-care system during the crisis. Infectious diseases in Indonesia continue to have a disparate impact on the health of Indonesians compared to many other low-income and middle-income countries. In the previous two decades, the government launched programmes to control tuberculosis, malaria, and other communicable diseases.^7^ Addressing the burden of non-communicable diseases, especially stroke, ischaemic heart disease, and diabetes, has likewise become prioritised by policy makers and civil society organisations. GBD 2019 estimates can be used to evaluate the overall impact of Indonesian health policy since 1990, and to set future directions for Indonesia and its provinces as the pandemic continues to unfold.

We previously published estimates of the burden of disease for Indonesia at the national level and evaluated the country's efforts to achieve UHC by 2020.^8^ Those national estimates provide an overview of the status of population health in Indonesia. For nearly a decade, GBD estimates have been used by Indonesian health officials and government agencies to assist in health policy decision making and budget allocation. These partnerships laid the groundwork for subnational analyses led by Indonesian researchers, policy makers, and government officials. The provincial estimates analysed in this paper are the next phase of this evolving project.

## Methods

### Overview

GBD 2019 estimated disease burden for 286 causes of death, 369 non-fatal causes of disability, and 87 risk factors for 204 countries and territories from 1990 to 2019. To generate estimates for Indonesia at the national level, we used 138 location-years of data to estimate Indonesia-specific demographic indicators, 317 location-years of data for Indonesia-specific causes of death, 689 location-years of data for Indonesia-specific non-fatal outcomes, 250 location-years of data for Indonesia-specific risk factors, and 1641 location-years of data for Indonesia-specific covariates. For the subnational level, we used 138 location-years of data to estimate Indonesia-specific demographic indicators, 5848 location-years of data for Indonesia-specific causes of death, 1534 location-years of data for Indonesia-specific non-fatal outcomes, 650 location-years of data for Indonesia-specific risk factors, and 16 016 location-years of data for Indonesia-specific covariates. GBD 2019 estimates for Indonesia included 1 915 207 total source metadata rows, and we used 821 total citations. Estimates of all-cause mortality, cause-specific mortality, years of life lost (YLLs), years lived with disability (YLDs), disability-adjusted life-years (DALYs), life expectancy at birth, healthy life expectancy (HALE), and related risk factors are reported between 1990 and 2019. Indonesian national estimates are equal to the sum of all subnational values. GBD 2019 complies with the Guidelines for Accurate and Transparent Health Estimates Reporting.^9^ All data sources used in the study are available on the Global Health Data Exchange website and a results query tool.

### Indonesian geographical units

We applied the standard GBD subnational estimation process to estimate all metrics by province of Indonesia from 1990 to 2019. To make accurate comparisons, data were adjusted to fit provincial and national boundaries for 2019 for the entire period. We modified the estimation process to account for changes to the national political map that occurred between 1990 and 2019. The former Indonesian province East Timor, which became the sovereign state of Timor-Leste in 2002, was excluded throughout the estimation process (appendix 2 pp 4–5).

### Estimation of mortality and causes of death

We estimated all-cause mortality for each year, sex, and location using GBD 2019 demographic methodology. This multistage process corrects biases from input data sources (eg, surveys, censuses, vital registration systems, sibling and birth histories, and household death recall) to estimate mortality rates for children younger than 5 years and adults using a combination of Gaussian processes and spatiotemporal regressions. This method is explained in detail elsewhere.^10^

Cause-specific mortality estimation for Indonesia required the standardisation of verbal autopsy, survey, and surveillance data to map deaths to aggregated causes in the GBD cause list. The most important source for cause-specific mortality in Indonesia was sample registration survey (SRS) verbal autopsies by province for all provinces in 2014 and 2015 and with partial coverage in 2013.^11^ We used SRS verbal autopsies because of the lack of a comprehensive death registration system.^12^ We used local verbal autopsies, sibling histories (for maternal deaths), and the National Socioeconomic Household Survey (Survei Sosial Ekonomi Nasional) done by Statistics Indonesia (Badan Pusat Statistik).^13^

In the case of insufficiently specific or implausible cause of death codes, we redistributed these so-called garbage codes using standard GBD algorithms and methods.^14^, ^15^ In most cases, we used cause of death ensemble modelling to estimate cause-specific mortality with information in the GBD cause of death database.^14^, ^15^ We calculated YLLs as the sum of each death multiplied by the reference standard life expectancy at each age.^14^, ^15^

We also calculated age-standardised YLL rates for Indonesia and all provinces in 2019 for the top 20 causes grouped by three levels of significance: significantly below the mean, indistinguishable from the mean, and significantly higher than the mean.

### Morbidity estimation

Non-fatal estimation uses diverse sets of data sources, including data from the GBD Collaborator Network, epidemiological surveillance data, disease registry data, systematic data and literature reviews, and hospital data to estimate prevalence, incidence, and other non-fatal outcomes using the Bayesian meta-regression tool DisMod-MR 2.0. YLDs were computed for each of the 369 non-fatal causes by multiplying the prevalence of non-fatal causes by the associated disability weight or combined disability weight for health states for each mutually exclusive sequela following comorbidity adjustment. Disability weight estimation^16^ and YLD computation^17^ are described in detail elsewhere.

### Combined health loss and healthy life expectancy

The sum of YLLs^14^ and YLDs^17^ for each location, year, age group, sex, and cause is equal to DALYs, a measure of overall health loss. DALYs combine both mortality and morbidity metrics to provide a standard metric to compare different causes of health loss. We used the Sullivan method to calculate HALE.^18^ Detailed methods of DALYs and HALE are available elsewhere.^19^

### Risk factors

GBD risk factors were organised hierarchically into three broad categories comprising behavioural, metabolic, and environmental and occupational risk factors. The disaggregation of these three types of risk factor into more refined categories allowed for comparisons at a lower level of abstraction. We used the GBD comparative risk assessment framework to estimate exposure to risk factors and deaths, as well as DALYs, by age, sex, location, and year.^20^ Additionally, for each risk, we produced a summary exposure value (SEV): a risk-weighted prevalence of an exposure. SEVs range from 0% to 100%, in which 0% reflected no risk exposure in a population and 100% indicated that an entire population was exposed to the maximum possible level for that risk. A detailed methodology on risk factors computation was previously published.^20^

### Decomposition of change

By adapting a method from Das Gupta,^21^ we decomposed the number of deaths by cause from 1990 to 2019 using population growth figures, age-based population changes, and shifts in cause-specific mortality rates. We used counterfactual scenarios to calculate the fraction of change in deaths by cause for each of the three components by changing the level of one component at a time and keeping the other two inputs constant during the entire period of study.

### Uncertainty analysis

We applied the technique for propagating uncertainty used for GBD 2019.^22^, ^23^, ^24^ We used 1000 draws by age, sex, location, and year in every step of the computation process to calculate the uncertainty interval (UI). To generate a 95% UI, we used the 2·5th and 97·5th percentiles. Calculations of point estimates used the means of the draws.^25^ A posterior probability of change of at least 95% defined statistically significant trends over time.

### The Socio-demographic Index

The Socio-demographic Index is a combined measure of development with a value between 0·0 and 1·0 calculated from the geometric mean of three rescaled components, comprising total fertility rate under age 25 years, lag-distributed income per capita, and average educational attainment in the population older than 15 years of age.^22^ The Socio-demographic Index correlates with health outcomes. It is an important metric because it allows for comparisons across geographies and regions. The 2019 Socio-demographic Index of Indonesia's 34 provinces ranged from 0·543 in East Nusa Tenggara to 0·802 in Jakarta. The Socio-demographic Index of the entire southeast Asia region is 0·644, which is lower than Indonesia's national Socio-demographic Index of 0·660.

### Role of the funding source

The funders had no role in study design, data collection, data analysis, data interpretation, or writing of this report.

## Results

### Life expectancy and healthy life expectancy

**Figure 1:**
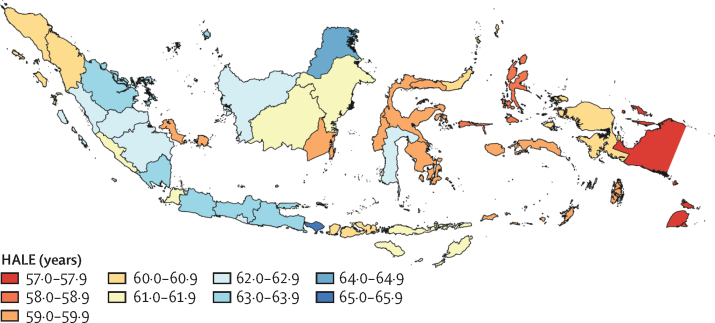
Map of variations in HALE by province for male an female sexes, 2019 HALE=healthy life expectancy.

For males, Bali had the highest life expectancy and HALE in 2019 and Papua had the lowest (table 1). For females, North Kalimantan had the highest life expectancy and HALE in 2019 and North Maluku had the lowest (table 2). Large disparities between provinces in life expectancy and HALE were also observed. For example, HALE for females differed by 10·4 years between the first-ranked and last-ranked provinces in 2019, whereas life expectancy differed by 13·7 years. These gaps, however, narrowed between 1990 and 2019. In 1990, HALE for females differed by 12·3 years between the first-ranked and last-ranked provinces and life expectancy differed by 17·3 years. We found a concentration of provinces with lower HALE for male and female sexes combined in eastern Indonesia, whereas provinces with higher HALE were in the western region (figure 1).

**Table 1:** Male life expectancy and healthy life expectancy at birth for Indonesia and its provinces, 1990 and 2019

	**Life expectancy at birth**				**Healthy life expectancy at birth**			
	1990		2019		1990		2019	
	Estimate (95% UI)	Rank	Estimate (95% UI)	Rank	Estimate (95% UI)	Rank	Estimate (95% UI)	Rank
Indonesia	62·5 (61·3–63·7)	..	69·4 (67·2–71·6)	..	55·5 (53·3–57·7)	..	61·2 (58·5–63·9)	..
Bali	65·5 (63·5–67·8)	4	74·4 (70·9–77·9)	1	56·7 (54·1–59·3)	6	64·0 (60·5–67·4)	1
Riau Islands	63·6 (61·3–66·4)	9	72·7 (69·7–76·2)	2	55·9 (53·3–58·7)	12	63·9 (60·8–67·3)	2
North Kalimantan	65·3 (62·4–68·3)	6	72·0 (68·5–75·8)	3	58·0 (54·9–61·0)	4	63·5 (60·0–67·0)	3
Jakarta	68·2 (66·0–70·5)	2	71·2 (68·2–74·4)	4	59·0 (56·2–61·8)	1	62·2 (59·1–65·4)	6
Central Java	66·3 (64·2–68·4)	3	71·0 (68·4–74·0)	5	58·0 (55·7–60·5)	3	62·2 (59·3–65·3)	7
Yogyakarta	68·9 (66·7–71·1)	1	70·7 (67·7–74·0)	6	58·7 (56·2–61·3)	2	61·6 (58·5–64·8)	13
Lampung	62·5 (60·4–64·7)	14	70·6 (67·4–74·1)	7	55·4 (52·8–58·1)	16	62·5 (59·4–65·8)	5
West Papua	65·5 (62·9–68·5)	5	70·6 (67·2–74·1)	8	57·4 (54·6–60·4)	5	62·1 (58·9–65·6)	9
West Java	62·2 (60·3–64·2)	15	70·5 (67·6–73·6)	9	55·7 (53·2–58·2)	14	62·2 (59·1–65·4)	8
Gorontalo	62·0 (59·6–64·5)	17	70·4 (66·9–74·1)	10	56·2 (53·6–59·2)	10	63·1 (59·7–66·6)	4
Riau	63·2 (61·1–65·3)	12	70·4 (67·2–73·8)	11	56·2 (53·6–58·8)	11	61·9 (58·7–65·1)	11
South Sumatra	63·5 (61·3–65·9)	10	70·2 (67·0–73·4)	12	56·4 (53·9–59·2)	7	62·1 (58·8–65·3)	10
West Kalimantan	61·0 (58·7–63·3)	25	70·0 (66·9–73·3)	13	54·4 (51·3–57·0)	24	61·4 (58·2–64·6)	14
Jambi	61·3 (59·0–63·6)	23	69·7 (66·8–72·9)	14	54·2 (51·6–56·7)	26	61·4 (58·2–64·5)	15
South Sulawesi	61·3 (59·0–63·7)	22	69·5 (66·6–72·6)	15	54·6 (52·0–57·4)	23	61·3 (58·0–64·4)	17
North Maluku	59·0 (56·4–61·9)	29	69·4 (65·9–72·9)	16	53·5 (50·7–56·4)	29	61·9 (58·5–65·5)	12
East Java	62·0 (60·1–63·9)	16	69·3 (66·5–72·3)	17	55·0 (52·5–57·3)	19	61·0 (58·0–64·0)	19
Bengkulu	61·4 (59·2–63·8)	21	69·3 (66·1–72·6)	18	55·2 (52·5–57·8)	17	61·3 (58·0–64·5)	16
Central Kalimantan	63·3 (61·0–65·7)	11	68·9 (66·0–72·1)	19	55·9 (53·4–58·7)	13	61·0 (58·0–64·2)	18
East Nusa Tenggara	59·2 (57·1–61·4)	27	68·7 (65·5–72·1)	20	53·6 (50·9–56·1)	28	60·9 (57·7–64·2)	20
West Sumatra	62·0 (59·8–64·4)	18	68·6 (65·7–72·0)	21	55·2 (52·9–57·7)	18	60·4 (57·2–63·7)	25
Aceh	64·2 (61·7–66·6)	7	68·6 (65·2–72·0)	22	56·4 (53·7–59·1)	8	60·0 (56·5–63·2)	28
Banten	59·1 (56·7–61·5)	28	68·5 (65·4–71·9)	23	54·2 (51·5–56·8)	25	60·9 (57·6–64·0)	21
East Kalimantan	64·2 (62·1–66·5)	8	68·4 (64·8–71·8)	24	56·3 (53·8–58·8)	9	59·9 (56·6–63·2)	30
North Sulawesi	62·9 (60·7–65·2)	13	68·3 (65·2–71·5)	25	54·9 (52·3–57·5)	20	60·6 (57·2–63·9)	22
West Nusa Tenggara	56·1 (53·8–58·5)	33	68·1 (64·6–71·5)	26	52·2 (49·6–54·7)	33	60·6 (57·3–64·0)	23
Central Sulawesi	57·8 (55·7–60·2)	30	67·5 (64·0–71·2)	27	52·4 (49·9–54·9)	31	60·3 (56·8–63·7)	26
Maluku	57·5 (55·0–60·3)	31	67·2 (63·7–71·1)	28	53·2 (50·4–56·0)	30	60·2 (56·8–63·6)	27
West Sulawesi	55·1 (52·9–57·7)	34	67·2 (63·7–70·7)	29	51·2 (48·7–53·8)	34	60·5 (57·1–64·0)	24
Southeast Sulawesi	61·9 (59·5–64·2)	19	67·1 (63·8–70·7)	30	55·5 (52·8–58·3)	15	59·9 (56·6–63·3)	29
Bangka-Belitung Islands	60·3 (57·8–62·9)	26	67·0 (63·6–70·5)	31	54·1 (51·5–56·8)	27	59·5 (56·1–62·9)	31
North Sumatra	61·6 (59·4–63·8)	20	66·3 (62·8–69·7)	32	54·8 (52·3–57·2)	22	59·3 (56·0–62·5)	32
South Kalimantan	56·4 (54·2–58·6)	32	65·4 (61·9–68·9)	33	52·2 (49·8–54·6)	32	58·6 (55·0–61·8)	33
Papua	61·2 (58·9–64·0)	24	64·5 (60·9–68·2)	34	54·9 (52·3–57·7)	21	58·3 (54·9–61·9)	34

UI=uncertainty interval.

**Table 2:** Female life expectancy and healthy life expectancy at birth for Indonesia and its provinces, 1990 and 2019

	**Life expectancy at birth**				**Healthy life expectancy at birth**			
	1990		2019		1990		2019	
	Estimate (95% UI)	Rank	Estimate (95% UI)	Rank	Estimate (95% UI)	Rank	Estimate (95% UI)	Rank
Indonesia	65·7 (64·5–s66·8)	..	73·5 (71·6–75·6)	..	56·5 (53·6–58·9)	..	62·9 (59·8–65·8)	..
North Kalimantan	70·8 (68·6–73·3)	1	77·7 (74·7–81·2)	1	60·8 (57·5–63·9)	1	66·6 (63·0–70·1)	1
Bali	67·8 (65·9–69·8)	5	76·5 (73·1–79·7)	2	57·1 (54·0–59·9)	8	64·4 (60·7–67·7)	2
East Java	67·5 (65·7–69·0)	6	75·3 (72·8–77·8)	3	57·9 (54·9–60·7)	4	64·3 (61·1–67·5)	3
Central Java	69·1 (67·5–70·9)	2	75·0 (72·6–77·5)	4	59·0 (56·0–61·7)	2	64·1 (60·7–67·1)	4
Jakarta	68·3 (66·3–70·2)	4	74·8 (72·1–77·3)	5	57·7 (54·6–60·6)	5	63·5 (60·1–66·8)	5
Riau	65·8 (63·0–67·3)	11	74·1 (71·2–76·7)	6	56·1 (53·0–58·7)	11	63·4 (60·0–66·6)	8
West Java	65·8 (64·1–67·5)	9	74·0 (71·4–76·4)	7	57·0 (54·0–59·9)	9	63·5 (60·2–66·7)	7
Lampung	67·2 (65·3–69·0)	7	73·8 (71·1–76·2)	8	58·0 (54·8–60·7)	3	63·5 (60·2–66·6)	6
West Sumatra	64·5 (62·5–66·7)	14	73·8 (71·1–76·8)	9	56·1 (53·2–59·1)	10	63·1 (59·8–66·2)	10
South Sumatra	66·7 (64·7–68·6)	8	73·2 (70·4–76·0)	10	57·4 (54·3–60·2)	6	63·2 (59·8–66·3)	9
South Sulawesi	63·2 (61·0–65·4)	19	73·2 (70·6–76·1)	11	55·1 (52·1–57·9)	16	62·9 (59·7–66·2)	11
Yogyakarta	68·6 (66·6–70·5)	3	73·2 (70·5–76·1)	12	57·3 (54·2–60·2)	7	62·4 (59·2–65·5)	13
Banten	63·7 (61·4–65·9)	17	72·5 (69·8–75·5)	13	55·8 (52·7–58·5)	14	62·3 (58·9–65·5)	14
Central Kalimantan	63·4 (61·3–65·7)	18	72·2 (69·5–75·2)	14	54·9 (52·0–57·7)	17	62·3 (58·8–65·2)	15
Riau Islands	65·0 (62·7–67·6)	12	72·2 (69·7–75·2)	15	55·7 (52·7–58·6)	15	61·9 (58·8–64·9)	17
West Kalimantan	60·1 (57·8–62·3)	23	72·0 (69·2–75·0)	16	52·5 (49·2–55·2)	24	61·7 (58·4–64·8)	19
North Sumatra	65·1 (63·0–67·2)	10	72·0 (69·2–74·9)	17	56·1 (53·1–58·7)	12	62·4 (58·9–65·6)	12
Jambi	60·8 (58·7–62·9)	21	71·8 (69·1–74·8)	18	53·2 (50·2–55·8)	21	61·9 (58·6–64·9)	16
East Kalimantan	63·8 (61·6–66·0)	16	71·6 (68·3–74·5)	19	54·6 (51·7–57·4)	18	61·0 (57·5–64·1)	21
East Nusa Tenggara	60·8 (58·5–63·1)	20	71·3 (68·3–74·6)	20	53·2 (50·4–56·1)	20	61·7 (58·3–65·0)	18
West Nusa Tenggara	57·2 (54·8–59·8)	31	70·7 (67·9–73·6)	21	51·4 (48·4–54·2)	27	61·1 (57·8–63·9)	20
Aceh	64·5 (62·2–66·8)	13	70·7 (67·7–73·9)	22	55·9 (52·7–58·8)	13	60·6 (57·4–63·8)	24
North Sulawesi	63·8 (61·5–66·3)	15	70·6 (67·9–74·0)	23	54·2 (51·0–57·1)	19	60·9 (57·5–64·2)	22
South Kalimantan	59·4 (57·3–61·8)	25	70·4 (67·3–73·2)	24	53·0 (50·3–55·5)	22	60·9 (57·4–63·9)	23
Bengkulu	59·3 (57·0–61·6)	26	70·1 (67·1–73·0)	25	52·0 (49·0–54·7)	25	60·5 (57·1–63·7)	25
Bangka-Belitung Islands	60·7 (58·3–63·1)	22	69·3 (66·2–72·5)	26	52·9 (49·9–55·8)	23	60·1 (56·9–63·1)	26
Southeast Sulawesi	57·7 (55·4–60·0)	29	68·9 (65·7–72·0)	27	51·0 (48·0–53·7)	30	59·9 (56·5–62·9)	27
Central Sulawesi	57·6 (55·4–60·0)	30	68·5 (65·4–71·9)	28	50·9 (48·0–53·8)	31	59·5 (56·3–62·6)	28
Maluku	57·1 (54·6–59·7)	32	67·2 (63·9–70·6)	29	50·8 (47·9–53·8)	32	58·4 (55·0–61·8)	30
West Sulawesi	53·5 (50·9–56·4)	34	67·0 (63·8–70·2)	30	48·5 (45·6–51·3)	34	58·7 (55·4–61·9)	29
West Papua	59·9 (57·2–62·7)	24	66·4 (62·6–69·8)	31	51·7 (48·7–54·6)	26	57·4 (53·7–60·7)	32
Papua	58·1 (55·8–60·6)	27	66·1 (62·9–69·5)	32	51·3 (48·3–53·9)	29	58·3 (54·9–61·7)	31
Gorontalo	58·1 (55·7–60·7)	28	65·0 (61·7–68·5)	33	51·4 (48·4–54·3)	28	57·1 (53·8–60·5)	33
North Maluku	54·9 (52·4–57·6)	33	64·0 (60·7–67·3)	34	49·0 (46·2–52·1)	33	56·2 (52·9–59·4)	34

UI=uncertainty interval.

### Years of life lost and years lived with disability

**Figure 2:**
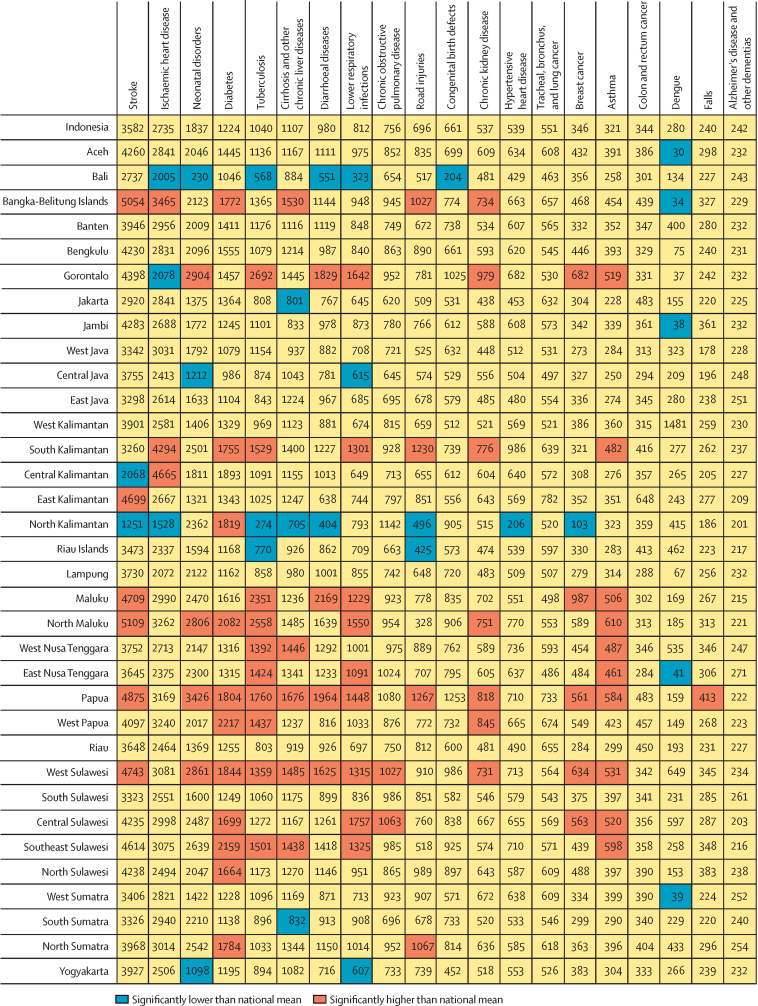
Age-standardised years of life lost (YLL) rates for Indonesia and all provinces for leading causes, 2019

Age-standardised death, YLL, and YLD rates varied widely between the provinces (table 3) but declined in all provinces except Aceh, which saw an increase of YLL rates per 100 000 between 1990 and 2019. Our analysis of the top 20 causes of YLL grouped by three levels of significance showed a clear pattern of performance, in which provinces were either lower or higher than the national mean across causes with some exceptions (figure 2). For example, West Sulawesi had an age-standardised YLL rate from chronic obstructive pulmonary disease higher than the mean, whereas North Kalimantan had a rate for road injuries significantly lower than the mean. There was little variation between provinces for Alzheimer's disease and other dementias, tracheal, bronchus, and lung cancer, colon and rectum cancer, or hypertensive heart disease (except for North Kalimantan).

**Table 3:** Age-standardised rates of death, YLL due to premature death, and YLD, Indonesia and provinces, 1990 and 2019

	**Age-standardised death rate per 100 000**				**Age-standardised YLL rate per 100 000**				**Age-standardised YLD rate per 100 000**			
	1990		2019		1990		2019		1990		2019	
	Rate (95%)	Rank	Rate (95%)	Rank	Rate (95%)	Rank	Rate (95%)	Rank	Rate (95%)	Rank	Rate (95%)	Rank
Indonesia	(1210 1150–1270)	..	951 (832–1020)	..	11 000 (8090–14 400)	..	10 300 (7600–13 400)	..	41 300 (39 300–43 300)	..	23 700 (20 900–26 300)	..
Aceh	1200 (1090–1330)	25	1060 (892–1190)	14	11 000 (8120–14 200)	24	11 300 (8410–14 400)	1	40 600 (36 800–44 600)	24	27 000 (23 000–31 300)	14
North Sumatra	1290 (1180–1400)	20	1070 (905–1220)	12	10 900 (8010–14 200)	27	10 300 (7570–13 300)	23	43 000 (39 500–46 600)	19	28 400 (24 100–33 100)	11
West Sumatra	1250 (1130–1370)	22	973 (831–1090)	22	10 900 (8030–14 200)	26	10 300 (7620–13 400)	21	43 500 (40 000–47 000)	18	23 700 (20 100–27 400)	22
Riau	1220 (1100–1330)	24	932 (771–1060)	24	10 800 (7950–14 000)	31	10 200 (7520–13 200)	28	41 100 (37 700–44 700)	23	22 100 (18 500–25 800)	28
Jambi	1440 (1300–1600)	11	1020 (837–1120)	20	11 200 (8200–14 600)	11	10 300 (7600–13 400)	22	47 800 (43 800–52 100)	15	24 800 (20 700–28 300)	20
South Sumatra	1150 (1050–1250)	30	931 (796–1080)	25	11 100 (8170–14 500)	14	10 200 (7600–13 300)	24	39 100 (35 900–42 600)	29	23 500 (20 100–27 200)	23
Bengkulu	1430 (1310–1590)	13	1060 (930–1180)	15	11 100 (8160–14 600)	13	10 400 (7680–13 600)	15	50 600 (46 800–55 200)	11	26 900 (23 100–31 000)	15
Lampung	1200 (1090–1310)	27	910 (764–1070)	29	11 100 (8140–14 500)	18	10 100 (7460–13 100)	32	39 900 (36 600–43 600)	26	22 600 (19 000–26 500)	25
Bangka-Belitung Islands	1470 (1320–1630)	7	1150 (1030–1310)	6	11 000 (8080–14 300)	25	10 200 (7560–13 300)	25	49 600 (45 000–54 200)	13	30 100 (26200–35 100)	9
Riau Islands	1240 (1110–1380)	23	912 (754–1030)	28	10 900 (8010–14 200)	30	10 200 (7540–13 200)	29	39 300 (35 500–43 200)	28	21 600 (18 300–24 800)	30
North Kalimantan	976 (852–1110)	34	737 (582–899)	33	10 600 (7850–13 800)	33	10 100 (7440–13 000)	33	34 300 (30 400–3 8700)	30	19 400 (16 000–23 300)	33
Jakarta	1020 (923–1120)	33	885 (741–1010)	31	10 900 (8040–14 300)	29	10 500 (7790–136 00)	10	32 200 (29 500–35 300)	33	20 700 (17 400–24 100)	31
West Java	1200 (1110–1280)	26	916 (779–1050)	27	11 100 (8160–14 500)	17	10 200 (7550–13 200)	26	42 000 (39 100–45 200)	20	22 200 (18 800–25 600)	26
Central Java	1030 (964–1110)	32	885 (749–984)	32	10 600 (7810–13 900)	34	10 100 (7490–13 200)	30	32 900 (30 300–35 800)	32	20 500 (17 500–23 600)	32
Yogyakarta	1090 (1010–1180)	31	930 (836–1030)	26	11 000 (8130–14 400)	22	10 400 (7710–13600)	14	29 300 (26 600–32 200)	34	22 200 (19 200–25 800)	27
East Java	1170 (1100–1260)	29	909 (786–1020)	30	11 100 (8130–14 600)	20	10 300 (7660–13 400)	20	39 500 (36 900–42 500)	27	22 000 (19 000–25 400)	29
Banten	1300 (1190–1420)	18	1020 (843–1140)	19	11 100 (8180–14 600)	12	10 300 (7670–13 400)	19	48 000 (44 100–52 500)	14	25 700 (21 500–29 500)	18
Bali	1190 (1080–1300)	28	728 (598–856)	34	10 900 (8050–14 200)	28	10 100 (7460–13 100)	34	34 100 (31 100–37 300)	31	16 300 (13 200–19 900)	34
West Nusa Tenggara	1610 (1450–1770)	5	1080 (943–1200)	11	11 100 (8200–14 500)	15	10 400 (7680–13 500)	17	61 100 (56 200–66 400)	2	27 600 (23 600–31 600)	13
East Nusa Tenggara	1460 (1320–1590)	9	1040 (857–1160)	17	11 300 (8280–14 700)	8	10 500 (7730–13 700)	11	51 700 (47 400–56 100)	9	265 00 (22 300–30 500)	16
West Kalimantan	1430 (1300–1570)	14	992 (874–1090)	21	11 500 (8410–16 100)	1	10 400 (7660–13 600)	16	50 000 (45 900–54 200)	12	24 100 (20 800–27 600)	21
Central Kalimantan	1300 (1170–1450)	19	1030 (859–1140)	18	10 700 (7840–13 900)	32	10 100 (7490–13 100)	31	41 900 (38 300–45 700)	22	25 400 (21 400–29 000)	19
South Kalimantan	1540 (1400–1680)	6	1160 (1020–1320)	5	11 000 (8140–14 500)	21	10 500 (7700–13 500)	13	57 700 (53 100–62 400)	5	30 800 (26 500–36 100)	6
East Kalimantan	1260 (1140–1400)	21	1050 (926–1200)	16	11 000 (8060–14 300)	23	10 600 (7810–13 800)	5	40 500 (37 000–44 100)	25	26 200 (22 500–30 900)	17
North Sulawesi	1370 (1230–1510)	15	1070 (894–1190)	13	11 500 (8440–14 800)	2	10 600 (7830–13 800)	6	42 000 (38 000–46 000)	21	27 600 (23 400–31 800)	12
Central Sulawesi	1640 (1490–1800)	3	1150 (978–1310)	7	11 400 (8370–14 900)	5	10 500 (7760–13 700)	9	57 700 (53 000–62 400)	6	30 600 (26 300–35 200)	8
South Sulawesi	1330 (1200–1450)	17	967 (806–1070)	23	11 100 (8180–14 500)	16	10 200 (7530–13 200)	27	45 900 (41 900–50 100)	16	23 400 (19 800–26 900)	24
Southeast Sulawesi	1460 (1330–1600)	8	1140 (960–1310)	9	11 400 (8400–14 900)	4	10 600 (7870–137 00)	4	52 600 (48 300–57 000)	8	30 800 (26 700–35 700)	7
Gorontalo	1430 (1300–1580)	12	1140 (915–1320)	8	11 300 (8310–14 700)	9	10 500 (7700–13 700)	12	54 200 (49 700–58 800)	7	32 200 (26 900–37 600)	5
West Sulawesi	1820 (1650–2010)	1	1180 (977–1350)	4	11 400 (8320–14 800)	6	10 400 (7660–13 400)	18	69 000 (62 700–753 00)	1	32 900 (28 100–38 000)	3
Maluku	1620 (1460–1790)	4	1200 (1040–1360)	3	11 100 (8170–14 400)	19	10 600 (7810–13 600)	8	59 700 (54 200–65 200)	3	32 300 (27 700–37 500)	4
North Maluku	1660 (1490–1860)	2	1210 (1030–1360)	2	11 400 (8410–14 900)	3	10 700 (7880–13 900)	2	59 300 (53 900–65 400)	4	33 800 (28 900–38 900)	2
West Papua	1370 (1190–1530)	16	1090 (946–1230)	10	11 200 (8210–14 500)	10	10 600 (7840–13 700)	7	44 500 (39 900–49 700)	17	29 000 (24 800–34 100)	10
Papua	1450 (1310–1600)	10	1280 (1050–1490)	1	11 300 (8290–14 700)	7	10 700 (7870–13 900)	3	51 400 (46 800–56 200)	10	37 300 (31 400–43 300)	1

YLD=years lived with a disability. YLL=years of life lost.

### Disability-adjusted life-years and risk factors

**Figure 3:**
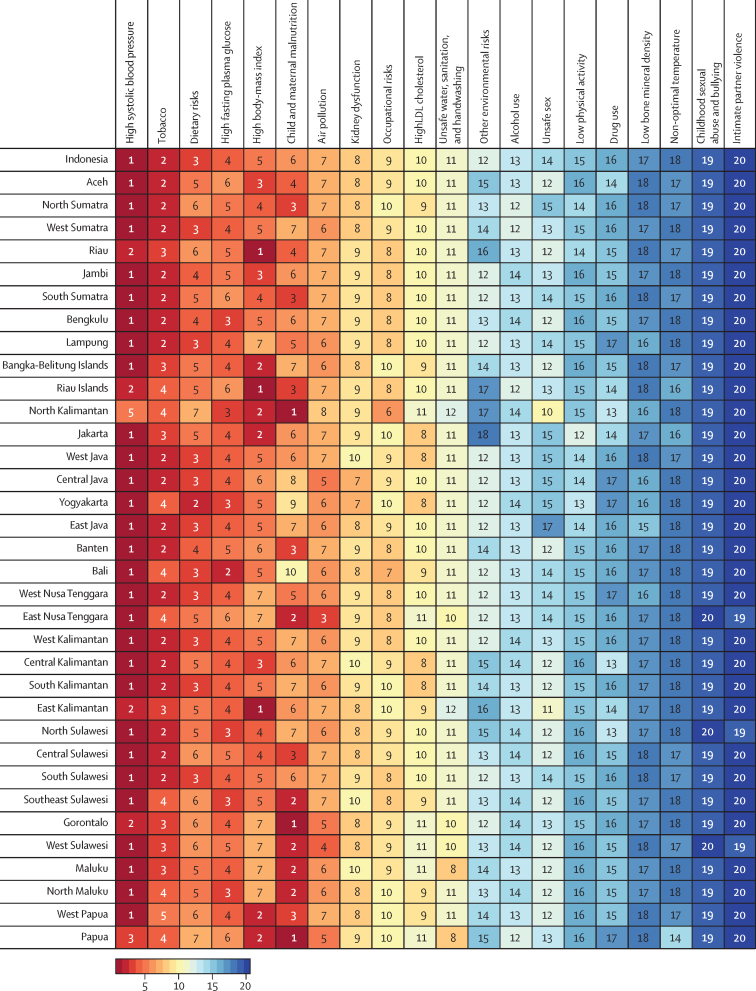
Leading risk factors for disability-adjusted life-years (DALYs) by province, 2019

The six leading risk factors for DALYs in Indonesia in 2019 were high systolic blood pressure, tobacco use, dietary risks, high fasting plasma glucose, high BMI, and child and maternal malnutrition (figure 3). High systolic blood pressure and tobacco use were among the top five leading risk factors for all provinces. Child and maternal malnutrition was the leading risk factor for North Kalimantan, Gorontalo, and Papua and the second-leading risk factor in East Nusa Tenggara, Southeast Sulawesi, West Sulawesi, Maluku, and North Maluku. High BMI was the leading risk factor for Riau, Riau Islands, and East Kalimantan and the second leading risk factor for Bangka-Belitung Islands, North Kalimantan, Jakarta, West Papua, and Papua.

## Discussion

This study provides a comprehensive assessment of the burden of diseases, injuries, and risk factors in Indonesia's provinces from 1990 to 2019. Our analysis provides valuable historical context for measuring and evaluating health progress in Indonesia during a three-decade period that has been defined by major administrative reforms and an ambitious push for UHC. Since 1990, health changes have been driven by an epidemiological transition of declining burden from infectious diseases and increasing burden from non-communicable diseases. Even while infectious diseases have decreased in their importance as leading causes of health loss in Indonesia, we found that the country maintains a double burden of disease. During the past 30 years, and since the country launched its UHC programme Badan Penyelenggara Jaminan Sosial Kesehatan (BPJS) in 2014,^26^ communicable diseases such as tuberculosis, diarrhoeal diseases, and lower respiratory infections have remained a main source of DALYs in Indonesia,^27^, ^28^, ^29^, ^30^, ^31^ whereas non-communicable diseases such as ischaemic heart disease and diabetes have soared. Effects of risk factors typically associated with diet and lifestyle constitute large shares of health loss in Indonesia. Child and maternal malnutrition is a considerable risk factor in several provinces, which suggests that reducing the burden of diet-based risk factors and diseases should be a priority for policy makers.^32^ Our findings reveal large disparities in health outcomes at the subnational level. GBD 2019 contains total estimates of health and health loss in Indonesia immediately before the COVID-19 pandemic, which sets a baseline for burden in each province. Some gains have been made in reducing the burden of communicable diseases and regional disparities since the implementation of BPJS in 2014. With prepandemic baselines, policy makers and stakeholders are equipped with information to evaluate the impact of policy interventions that might otherwise be obscured or distorted by COVID-19.

Indonesia's epidemiological transition continues to unfold. Reductions in the communicable disease burden have been slow, while non-communicable diseases continue to affect Indonesian health, albeit in uneven patterns across the provinces. The high burden of non-communicable diseases in the provinces with high Socio-demographic Index requires further attention from policy makers and stakeholders. Non-communicable diseases such as diabetes are urgent health policy concerns (appendix 2 pp 9–10). Diabetes is a particularly expensive disease to treat and manage.^33^ The Indonesian Government has launched national campaigns against hypertension, diabetes, and obesity. For instance, Gerakan Indonesia Lawan Diabetes is an initiative launched by the Ministry of Health in collaboration with PT Kalbe Farma, a large health-care provider and pharmaceutical company, that raises awareness about diabetes prevention and treatment. The National Health Social Security Agency (BPJS Kesehatan) initiated the Chronic Disease Management Program (Program Pengelolaan Penyakit Kronis, PROLANIS) in 2010.^34^

Indonesia invested US$348 million between 2003 and 2017 to procure drugs, bednets, and tests for malaria with the assistance of the Global Fund to Fight AIDS, Tuberculosis and Malaria.^35^ Another communicable disease that has received attention from policy makers, researchers, and practitioners is tuberculosis (appendix 2 p 9).^36^, ^37^, ^38^ The National TB Control Program provides policy guidance and oversight of Indonesia's goal to eliminate tuberculosis by 2035. Continuing to invest in programmes that address the high burden of communicable diseases in Indonesia by using subnational estimates will not only help address provincial and regional health disparities, but also lift the overall health profile of the country.

This study shows that high systolic blood pressure, high BMI, smoking, poor diet, and high fasting plasma glucose are the five largest risks in Indonesia. These five risks threaten to stall or even reverse health gains in Indonesia and have the potential to redirect the future health trajectory for many provinces. It is imperative that policies and interventions designed to decrease the burden of disease through reductions in modifiable dietary, metabolic, and other risk factors are prioritised at each administrative level. Indonesia is also trying to control tobacco use through various mechanisms and regulations.^28^, ^29^, ^30^ This multipronged approach to reducing tobacco consumption in Indonesia, however, has not had the effect on the scale that is needed, and it is time for a more robust effort. Indonesia has not signed the WHO Framework Convention on Tobacco Control despite its high tobacco burden and increased pressure to join the global community and ratify the agreement. Ratifying this agreement would reinforce the domestic programmes already implemented.^39^, ^40^ Indeed, Indonesia needs to invest broadly in behavioural change, prevention, and health promotion; however, for interventions to succeed, a multisectoral approach is needed. Involvement of local communities is essential. Successful efforts on this front are underway. For example, the Gerakan Masyarakat Hidup Sehat is a movement for healthy living that has received presidential support in Indonesia and illustrates the efficacy of coordinated public health campaigns. Additionally, strengthening Indonesia's community-based health network Puskesmas could help address risk factors and preventable diseases in rural and underserved areas. Increasing the availability of medical devices or providing enhanced training and recruitment of community health workers through Puskesmas, for example, could have positive effects for populations in remote areas of the country.

Our analysis reveals substantial health inequalities across provinces. Western provinces have higher ranks overall in health development indices compared to eastern regions.^4^, ^31^ North Kalimantan, Bali, Jakarta, and other western provinces ranked consistently high on all indicators, whereas eastern provinces, including Papua and North Maluku, tended to appear near the bottom in most categories. For example, according to Healthcare Access and Quality (HAQ) Index estimates, the 14 provinces that had an HAQ Index higher than the national mean value of Indonesia were largely in the western part of the country, whereas the remaining 20 provinces that had HAQ Index values lower than the national estimate are mostly concentrated in the east (appendix 2 p 9).^41^ HALE for male and female sexes in the two highest-performing provinces, Bali and North Kalimantan, was 65·8 and 64·9 years, respectively. HALE in the two lowest-performing provinces, Papua and North Maluku, was 57·3 and 58·5, respectively—a difference of 8·5 years between Bali and North Kalimantan and 6·4 years between Papua and North Maluku. Likewise, average life expectancy for the three highest-ranking provinces (Bali, North Kalimantan, and Jakarta) was 74·26—or 8·02 more years of life expectancy than the three lowest-ranking provinces (Papua, North Maluku, and West Sulawesi), which have an average life expectancy of 66·24 years. The accuracy and usefulness of estimates of health and health loss for each province in Indonesia could be enhanced by generating evidence at the district and municipal levels. A look at health variations within a province at finer geospatial levels is necessary to identify hot spots for targeted interventions.^42^, ^43^, ^44^

On the basis of our analysis, we offer several policy recommendations that we believe could have a positive effect on health and health outcomes across provinces. The burden of non-communicable diseases, especially in provinces with high Socio-demographic Index, requires special attention from health authorities and can be achieved through reductions in modifiable dietary, metabolic, and other risk factors. Indonesia must also continue to invest in programmes that address the high burden of communicable diseases, such as tuberculosis. Strengthening Puskesmas and other community-based health programmes could help improve health outcomes in rural and underserved areas. Indonesia must also better integrate its policies at all levels of government and strive for synergism between the central government and service-providing agencies at the provincial and district levels to hit targets and close gaps in health outcomes. Integrating health information systems, improving monitoring of health inequality, and increased health spending for and by provincial governments are all areas that could help achieve policy alignment. Policies and interventions meant to address regional health inequalities in Indonesia would benefit from at least three approaches: addressing key modifiable risks, including diet, smoking, and high BMI; improving access to high-quality health care in underserved urban and rural areas; and addressing the social determinants of health. These three strategies will need to vary by region and province considering the country's geographical diversity, sociocultural connectivity, fiscal realities, and infrastructure. Improving the quality, reliability, and availability of data at all administrative levels of Indonesia, especially at the provincial and subprovincial levels, will assist in the generation and analysis of useful health metrics that could be used to address regional inequalities. GBD estimates can augment existing health metrics resources, such as the Indonesian Basic Health Research reported by the Ministry of Health, by providing deep historical context and specialised metrics that are comparable across locations.^45^, ^46^ Included in these efforts could also be improved disease surveillance systems and a focus on pandemic preparedness.

GBD 2019 can serve as a baseline for tracking future health trends as Indonesia continues to battle COVID-19. Indonesia has been a regional epicentre of the coronavirus pandemic in southeast Asia. Vaccine production and vaccination continue as authorities try to meet the benchmarks set by the ambitious plan rolled out by the central government. The government has responded to the economic fallout from the virus through a series of reform bills and stimulus programmes. The long-term health consequences of the pandemic are not yet known. From immunisations to preventive services, the pandemic will have a significant impact on Indonesia's health-care system, and GBD 2019 allows us to track these developments over time. Future GBD data will enable health officials at all levels of administration to evaluate the performance of health systems in Indonesia before the onset of the pandemic and after.

Given the scope of our analysis, this study has several limitations. The overall limitations of the GBD methods as noted in other publications apply to this Indonesia analysis.^17^, ^18^, ^19^ The accuracy of the estimates depends on the availability of data by period. Second, it is challenging to separate measurement error from variation in disease occurrence. GBD corrects for known bias from non-reference methods or case definitions, but often must rely on sparse data to make those adjustments. Third, GBD includes risk–outcome pairs that meet the World Cancer Research Fund criteria of causality. However, some risk–outcome pairs might not meet criteria that developed as evidence from new studies are published. Fourth, some of the data used in the analyses has a lower quality and consistency across sex and age groups. GBD 2019 reports 95% UIs to provide the effect of this limitation on the estimates.

The study revealed large disparities in the burden of disease among provinces in Indonesia. Although some of these disparities are expected because of differences in socioeconomic status, resources, and geography of the provinces, the variable rates of health gains and losses for many provinces is a concern. Our findings contribute to the development and implementation of provincial health plans for future planning cycles to address the leading challenges and ensure health equity during the current pandemic and beyond. Empirical studies in several health and scientific fields will complement GBD and help shore up the evidence base for health experts and policy decision makers.

## Data sharing

This paper summarises key findings from our analysis of GBD 2019 estimates. All subnational estimates are publicly available in our online tools (http://ghdx.healthdata.org/gbd-2019). Citations for the data used in this study can be accessed from the Global Health Data Exchange data input sources tool (http://ghdx.healthdata.org/gbd-2019/data-input-sources). Files containing all GBD 2019 subnational estimates are available on the Global Health Data Exchange website (http://ghdx.healthdata.org/gbd-2019) and can also be downloaded from the Global Health Data Exchange results tool (http://healthdata.org/gbd-results-tool). Additional results can be explored through online interactive visualisations (https://vizhub.healthdata.org/gbd-compare/).

## Supplementary Material

Bahasa Indonesia translation of the abstract
