# Correction to *Lancet Glob Health* 2022; 10: e1632–45

**DOI:** 10.1016/S2214-109X(23)00174-2

**Published:** 2023-04-07

*GBD 2019 Indonesia Subnational Collaborators. The state of health in Indonesia's provinces, 1990–2019: a systematic analysis for the Global Burden of Disease Study 2019.* Lancet Glob Health *2022;* 10: *e1632–45*—For this Article, The GBD 2019 Indonesia Subnational Collaborators list and appendix 2 have been updated. In addition, the appendix page numbers in the main text have been updated. This correction has been made as of April 7, 2023.

