# Supplementary material for: The state of health in Indonesia's provinces, 1990–2019: a systematic analysis for the Global Burden of Disease Study 2019
Source: Lancet Glob Health. 2022 Oct 11;10(11):e1632–45. doi: 10.1016/S2214-109X(22)00371-0 (PMC9579357; doi:10.1016/S2214-109X(22)00371-0)
Supplement: Bahasa Indonesia translation of the abstract [file mmc1.pdf]

THE LANCET
Global Health

Supplementary appendix 1

This translation in Bahasa was submitted by the authors and we reproduce it as supplied. It has not been peer reviewed. *The Lancet*'s editorial processes have only been applied to the original in English, which should serve as reference for this manuscript.

Terjemahan dalam Bahasa Indonesia ini diserahkan oleh penulis dan diproduksi kembali apa adanya tanpa melalui proses peer review. Proses editorial Lancet hanya diterapkan pada versi original dalam Bahasa Inggris, yang harus dijadikan rujukan untuk naskah ini.

Supplement to: GBD 2019 Indonesia Subnational Collaborators. The state of health in Indonesia's provinces, 1990–2019: a systematic analysis for the Global Burden of Disease Study 2019. *Lancet Glob Health* 2022; **10:** e1632–45.

Abstrak

Latar Belakang
Menganalisa tren dan tingkat beban penyakit di tingkat nasional seringkali dapat menutupi ketidaksetaraan dalam kemajuan terkait kesehatan di unit administratif yang lebih rendah, seperti provinsi dan kabupaten. Kami menggunakan hasil Studi Global Burden of Disease 2019 (GBD 2019) untuk menganalisa pola kesehatan di Republik Indonesia di tingkat provinsi antara tahun 1990 dan 2019. Analisis beban penyakit jangka panjang memberikan wawasan tentang perjalanan Indonesia menuju Jaminan Kesehatan Semesta dan kemampuan untuk memenuhi Tujuan Pembangunan Berkelanjutan PBB pada tahun 2030.

Metode
Kami menganalisa GBD 2019 untuk perkiraan kematian menurut penyebab tertentu, tahun hidup yang hilang akibat kematian dini (YLLs), tahun hidup dengan disabilitas (YLDs), tahun hidup sehat yang hilang (DALYs), usia harapan hidup saat lahir, usia harapan hidup sehat (HALE), dan faktor risiko untuk 286 penyebab kematian dan 369 penyebab kehilangan kesehatan non-fatal berdasarkan tahun, usia, dan jenis kelamin untuk Indonesia dan 34 provinsi dari tahun 1990 hingga 2019. Untuk menghasilkan estimasi di tingkat nasional untuk Indonesia, kami menggunakan 138 data lokasi-tahun untuk memperkirakan indikator demografi spesifik untuk Indonesia, 317 data lokasi-tahun untuk penyebab kematian spesifik untuk Indonesia, 689 data lokasi-tahun untuk hasil non-fatal spesifik untuk Indonesia, 250 data lokasi-tahun untuk faktor risiko spesifik untuk Indonesia, dan 1.641 data lokasi-tahun untuk kovariat khusus untuk Indonesia. Untuk perkiraan subnasional, kami menggunakan sumber hitungan sebagai berikut: 138 data lokasi-tahun untuk memperkirakan indikator demografi spesifik untuk Indonesia, 5.848 data lokasi-tahun untuk penyebab kematian spesifik untuk Indonesia, 1.534 data lokasi-tahun untuk hasil non-fatal spesifik untuk Indonesia, 650 data lokasi-tahun untuk faktor risiko spesifik untuk Indonesia, dan 16.016 data lokasi-tahun untuk kovariat spesifik untuk Indonesia. Kami membuat perkiraan GBD 2019 untuk Indonesia dengan menggunakan total 1.915.207 baris metadata sumber, dan kami menggunakan total 821 kutipan.

Temuan

Ada kesenjangan yang besar dalam hasil kesehatan antar provinsi. Untuk laki-laki dan wanita di seluruh Indonesia, Bali memiliki usia harapan hidup saat lahir tertinggi pada tahun 2019 yaitu 75,4 tahun (95% UI 73,1–77,8), sedangkan Papua memiliki usia harapan hidup terendah pada 65,2 (62,7– 67,9), dengan rentang perbedaan 10,2 tahun. Pada tahun 2019, Bali juga memiliki HALE saat lahir tertinggi, yaitu 65,8 tahun (62,7–68,6), sedangkan Papua memiliki HALE terendah pada 57,3 tahun (54,3–60,2), dengan rentang perbedaan sebesar 8,5 tahun. Usia harapan hidup laki-laki di seluruh Indonesia meningkat dari 62,5 (61,3–63,7) menjadi 69,4 (67,2–71,6) antara tahun 1990 dan 2019, perubahan positif sebesar 6,9 tahun. Untuk wanita selama periode yang sama, usia harapan hidup meningkat dari 65,7 (64,5-66,8) menjadi 73,5 (71,6-75,6), meningkat 7,8 tahun. Peluang kematian ketika lahir hingga usia 20 tahun dan dari 20 tahun hingga 55 tahun menurun di semua provinsi untuk kedua jenis kelamin, tetapi meningkat untuk usia 55 tahun hingga 90 tahun di Papua, Maluku Utara, Papua Barat, Aceh, Kalimantan Timur, dan Banten. Tingkat kematian berbaku usia, YLL, dan YLD juga sangat bervariasi antar provinsi pada tahun 2019. Tekanan darah sistolik tinggi, tembakau, risiko diet, glukosa plasma puasa tinggi, dan indeks massa tubuh tinggi adalah lima risiko utama berkontribusi terhadap hilangnya kesehatan yang diukur melalui DALY di tahun 2019

Kesimpulan

Temuan kami menyoroti bahwa Indonesia menghadapi “beban ganda” penyakit menular dan tidak menular yang bervariasi antar provinsi. Dari tahun 1990 hingga 2019, Indonesia mengalami penurunan beban penyakit menular yang mendorong meningkatnya kesehatan di seluruh wilayah, meskipun penyakit menular seperti TB, penyakit diare, dan infeksi saluran pernapasan bawah tetap menjadi beberapa sumber utama DALY di Indonesia. Namun, selama periode yang sama, tingkat kematian dan disabilitas semua usia akibat penyakit tidak menular, serta paparan terhadap faktor risiko terkait, berkontribusi lebih besar terhadap hilangnya kesehatan. Perbedaan hasil kesehatan antara provinsi yang memiliki kinerja tertinggi dan terendah juga melebar sejak tahun 1990. Temuan kami mendukung adanya proses komprehensif untuk meninjau kembali kebijakan kesehatan yang ada saat ini, memeriksa penyebab-penyebab timbulnya variasi beban penyakit antar provinsi, dan memperkuat program dan kebijakan yang ditujukan untuk mengurangi kesenjangan di seluruh negeri. Indonesia terus membuat kemajuan dalam mencapai pelayanan kesehatan universal.
