# Supplementary material for: The state of health in Indonesia's provinces, 1990–2019: a systematic analysis for the Global Burden of Disease Study 2019
Source: Lancet Glob Health. 2022 Oct 11;10(11):e1632–45. doi: 10.1016/S2214-109X(22)00371-0 (PMC9579357; doi:10.1016/S2214-109X(22)00371-0)
Supplement: Supplementary appendix [file mmc2.pdf]

THE LANCET
Global Health

Supplementary appendix 2

This appendix formed part of the original submission and has been peer reviewed. We post it as supplied by the authors.

Supplement to: GBD 2019 Indonesia Subnational Collaborators. The state of health in Indonesia's provinces, 1990–2019: a systematic analysis for the Global Burden of Disease Study 2019. *Lancet Glob Health* 2022; **10:** e1632–45.

Supplementary appendix to “The state of health of Indonesia’s provinces, 1990–2019: a systematic analysis for the Global Burden of Disease Study 2019”

This appendix provides supplementary figures and more detailed results for “The state of health of Indonesia’s provinces, 1990–2019: a systematic analysis for the Global Burden of Disease Study 2019.”

Table of Contents

Section 1: Abbreviations ***3***

Section 2: GATHER compliance ***3***

Section 3: Locations and subnational geographical units ***3***

Section 3a: Indonesian provinces and provincial capital cities **3**

Section 3b: Indonesian subnational geographical units **4**

Section 3c: National and subnational source counts **5**

Section 4: Regulations on decentralisation in Indonesia, 1999–2018 ..... ***5***

Section 5: Age-based health disparities ***6***

Section 6: Future health scenarios ***6***

Section 7: Supplementary tables ***6***

Table S1. Leading causes of death and premature mortality **6**

Table S2. Leading causes of years lived with disability **6**

Table S3. Life expectancy and healthy life expectancy for males and females combined in Indonesian provinces, 1990-2019 ..... **7**

Table S4. Changes in age-standardised summary exposure values for leading risk factors **7**

Table S5. SDI values for Indonesia and 34 provinces between 1990 and 2019 ..... **7**

Table S6. Healthcare Access and Quality Index for Indonesian subnational units **7**

Table S7. Burden of lower respiratory infections, 1990–2019 ..... **7**

Table S8. Under-5 diarrhoeal and lower respiratory illnesses by province **7**

Section 8: Supplementary figures ***8***

Figure S1. Leading causes of disability-adjusted life-years **8**

Figure S2. Attribution of disability-adjusted life-years to risk factors **8**

Figure S3. Deaths and disability-adjusted life-years from leading risk factors **8**

Figure S4. Decomposition of the probability of death by age and province **8**

Figure S5. Map of Healthcare Access and Quality Index in Indonesia **9**

Figure S6. Road traffic deaths rates and percentage changes in Indonesia, 1990–2019 ..... **9**

Figure S7. Age-standardised disability-adjusted life-years estimates for diarrhoea 9
Section 9: Additional insights *9*
Section 9a: The National Tuberculosis Control Program 9
Section 9b: Non-communicable diseases in Indonesia... 9
Section 10: Contributions *10*
Section 11: GATHER table *11*
GATHER Checklist 11

Section 1: Abbreviations

**Abbreviation**	**Full phrase**
BPJS *Kesehatan*	*Badan Penyelenggara Jaminan Sosial Kesehatan*
CODEm	Cause of Death Ensemble modelling
COPD	chronic obstructive pulmonary disease
COVID-19	coronavirus disease 2019
DALY	disability-adjusted life-years
GATHER	Guidelines for Accurate and Transparent Health Estimates Reporting
GBD	Global Burden of Diseases, Injuries, and Risk Factors Study
GERMAS	*Gerakan Masyarakat Hidup Sehat*
GILD	*Gerakan Indonesia Lawan Diabetes*
HALE	healthy life expectancy
HAQ	Healthcare Access and Quality
IHD	ischaemic heart disease
IHME	Institute for Health Metrics and Evaluation
JKM	Community Health Insurance (*Jaminan Kesehatan Masyarakat*)
JKN	National Health Insurance scheme (*Jaminan Kesehatan Nasional*)
JKS	Social Health Insurance (*Jaminan Kesehatan Sosial*)
LE	life expectancy
LMIC	low- and middle-income countries
PROLANIS	Chronic Disease Management Program (*Program Pengelolaan Penyakit Kronis*)
SARS-CoV-2	severe acute respiratory syndrome coronavirus 2
SDI	Socio-demographic Index
SDG	Sustainable Development Goals
SEV	summary exposure value
SRS	sample registration survey
TB	tuberculosis
UHC	universal health coverage
U5M	under-5 mortality
UI	uncertainty interval
WHO	World Health Organization
YLD	years lived with disability
YLL	years of life lost

Section 2: GATHER compliance

This study complies with GATHER recommendations. We have documented the steps in our analytical procedures and detailed the data sources used. See section 10 for the GATHER checklist. The GATHER recommendations can be found on the GATHER website.

Section 3: Locations and subnational geographical units

Section 3a: Indonesian provinces and provincial capital cities

Province name	Capital
Aceh	Banda Aceh
Bali	Denpasar
Bangka Belitung Islands	Pangkalpinang
Banten	Serang

Bengkulu	Bengkulu
Central Java	Semarang
Central Kalimantan	Palangka Raya
Central Sulawesi	Palu
East Java	Surabaya
East Kalimantan	Samarinda
East Nusa Tenggara	Kupang
Gorontalo	Gorontalo
Jakarta	Jakarta
Jambi	Jambi
Lampung	Bandar Lampung
Maluku	Ambon
North Kalimantan	Tanjung Selor
North Maluku	Sofifi
North Sulawesi	Manado
North Sumatra	Medan
Papua	Jayapura
Riau	Pekanbaru
Riau Islands	Tanjungpinang
South Kalimantan	Banjarmasin
South Sulawesi	Makassar
South Sumatra	Palembang
Southeast Sulawesi	Kendari
West Java	Bandung
West Kalimantan	Pontianak
West Nusa Tenggara	Mataram
West Papua	Manokwari
West Sulawesi	Mamuju
West Sumatra	Padang
Yogyakarta	Yogyakarta

Section 3b: Indonesian subnational geographical units

The standard GBD subnational estimation process was used to estimate all metrics by province of Indonesia from 1990 to 2019. To make accurate comparisons, data are adjusted to fit provincial and national boundaries for 2019 for the entire period. We modified the estimation process to account for major changes to the national political map that occurred between 1990 and 2019. During the period of study (1990–2019), eight new provinces were formed in Indonesia and one former province became an independent state. In 1999, West Irian Jaya (present-day West Papua) and North Maluku were formed from Irian Jaya and Maluku, respectively. Three new provinces were formed in 2000: Banten, Bangka Belitung Islands, and Gorontolo. In 2002, Riau Islands was formed from Riau; West Sulawesi became a province in 2004. Most recently, in 2012, North Kalimantan was formed from East Kalimantan. The former Indonesian province East Timor, which became the sovereign state of Timor-Leste in 2002, was excluded throughout the estimation process. Data from the entire period of study were adjusted to fit present-day national and provincial units.

As noted in the main manuscript, there are three administrative levels to Indonesia's government: the central, provincial, and district or municipal governments. It is important to understand

the relationship between these three levels of government vis-à-vis the country's health system. In this context, the use of "subnational" has two meanings. First, subnational refers to the provincial level of government; it also refers to the district and municipal governments at the lower level of the country's administrative order. Indonesia started a decentralisation process in 1999. Responsibility for many fields of governance and policy implementation, including health, was devolved to districts and municipalities. In accordance with Government Regulation 33 in 2018, provincial governors were vested with the authority to serve as representatives of the central government. In this capacity, governors were charged with the responsibility to coordinate and assess development activity. Importantly, Regulation 33 did not change the responsibilities or status of districts and municipalities.

Section 3c: National and subnational source counts

To generate estimates for Indonesia at the national level, we used 138 location-years of data to estimate Indonesia-specific demographic indicators, 317 location-years of data for Indonesia-specific causes of death, 689 location-years of data for Indonesia-specific non-fatal outcomes, 250 location-years of data for Indonesia-specific risk factors, and 1641 location-years of data for Indonesia-specific covariates. For subnational estimates, we used the following source counts: 138 location-years of data to estimate Indonesia-specific demographic indicators, 5848 location-years of data for Indonesia-specific causes of death, 1534 location-years of data for Indonesia-specific non-fatal outcomes, 650 location-years of data for Indonesia-specific risk factors, and 16 016 location-years of data for Indonesia-specific covariates. For GBD 2019 estimates for Indonesia, we analysed 1 915 207 total source metadata rows, and used 821 total citations. For a complete list of all data sources used in our analysis, please consult the Global Health Data Exchange (GHDx)[http://ghdx.healthdata.org] and the GBD 2019 Data Input Services Tool [http://ghdx.healthdata.org/gbd-2019/data-input-sources].

Section 4: Regulations on decentralisation in Indonesia, 1999–2018

Key national regulations on decentralisation in Indonesia, 1999–2018

Number	Ttg	Regarding	Pres<br>Date
UU No 22 thn 1999	Pemerintahan Daerah	Local Government	B.J. Habibie<br>7 May 1999
UU No 32 thn 2004	Pemerintahan Daerah	Local Government	Megawati Sukarnoputri<br>15 October 2004
UU No 23 thn 2014	Pemerintahan Daerah	Local Government	Susilo Bambang Yudhoyono<br>2 October 2014
PP No 33 thn 2018	Pelaksanaan Tugas dan Wewenang Gubernur Sebagai Wakil Pemerintah Puast	Implementation of Responsibilities & Authority of Governor as Representative of the National Government	Joko Widodo<br>20 July 2018

Section 5: Age-based health disparities

Our analysis throws age-based health disparities into sharp relief. Even as Indonesia becomes an increasingly young country by global standards, health officials and policy makers must prepare for the unique challenges of caring for an ageing population. Life expectancy for men ages 62 to 69 and women ages 65 to 74 both increased between 1990 and 2019. Geographical variations in health-care access for aged populations, however, make a one-size-fits-all solution unfeasible. Elderly urban people have been observed with a higher probability of outpatient use than elderly rural people. Elderly with less education were more likely to be outpatients at primary health-care centres than elderly with higher education. Analysis of subnational data related to under-5 mortality (U5M) found patterns of higher mortality in more recently formed administrative regions, islands, and isolated areas, which suggests a critical need for more attention and resources for those areas.

Section 6: Future health scenarios

To be better prepared for the global health challenges ahead, IHME reported future health scenarios for 250 causes of death through 2040 in 195 countries. Health officials in Indonesia will need to examine the future health scenarios for each province and invest in strategies that are tailored to each situation to reduce future burden. For more information about IHME's future health scenarios, see Foreman KJ, Marquez N, Dolgert A, *et al.* Forecasting life expectancy, years of life lost, and all-cause and cause-specific mortality for 250 causes of death: reference and alternative scenarios for 2016–40 for 195 countries and territories. *Lancet* 2018; 392: 2052–90.

Section 7: Supplementary tables

Table S1. Leading causes of death and premature mortality

Supplementary Table 1 lists the 25 leading causes of death and years of lost life (YLLs) from 1990 to 2019. We report 95% uncertainty intervals for each disease and injury. Stroke and IHD were the first and second leading causes of death and YLLs in 2019. Of the top 25 leading causes of YLLs, age-standardised YLLs rates increased for only six causes (tracheal, bronchus, and lung cancer; breast cancer; colon and rectum cancer; IHD; diabetes). Table 1 highlights declines by more than half of age-standardised death rates for tuberculosis, diarrhoeal diseases, lower respiratory infections, neonatal disorders, dengue, protein-energy malnutrition, congenital birth defects, and typhoid and paratyphoid. Table 1 also shows changes in the leading causes of YLLs and death from 1990 to 2019. The results show a noticeable decline in infectious diseases alongside a rise in non-communicable diseases. For example, between 1990 and 2019, YLLs (in thousands) due to type 2 diabetes increased from 1010 (95% UI 890–1130) to 2950 (2480–3480). Deaths (in thousands) due to type 2 diabetes during that same period increased from 33·2 (29·6–37·3) to 106 (90–122). The mean percentage change of type 2 diabetes for the age-standardised YLL rate increased by 41·0% (15·1–69·4) and increased by 50·1% (25·2–76·2) for age-standardised death rate. Lower respiratory infections decreased, with a 75·0% (68·3–81·1) decline in age-standardised YLL rates and a 51·0% (41·3–58·8) decline in age-standardised death rate.

Table S2. Leading causes of years lived with disability

Supplementary Table 2 provides the 25 leading diseases and injuries contributing to YLDs. The first and second causes of YLDs in 2019 – low back pain and headache disorders – are the same as in 1990. YLDs for seven non-communicable diseases were found to have increased more than 100% since 1990: neonatal disorders (234% [95% UI 160–344]), stroke (118% [112–125]), chronic obstructive pulmonary

disease (COPD) (103% [98·3–108)], diabetes (250% (236–263)], chronic kidney disease (148% [131–165)], osteoarthritis (150% [145–154]), and other musculoskeletal disorders (150% [139–164]). Notably, YLDs for lower back pain and neck pain have increased 93.9% (83·4–102) and 71.4% (65·6–77·5), respectively, since 1990. In 2019, type 2 diabetes was the tenth leading cause of YLDs and had a 61·5% (55·9–67·0) increase in age-standardised rates from 1990, when it was ranked 20th. Dietary iron deficiency had a YLD rank of third in 1990 and 11th in 2019 and declined 46·0% (37·0–54.0) in age-standardised rates.

Table S3. Life expectancy and healthy life expectancy for males and females combined in Indonesian provinces, 1990-2019

Supplementary Table 3 shows life expectancy and HALE at birth for Indonesia and the 34 provinces for both sexes combined. Bali had the highest life expectancy (75·4 [73·1–77·8]) and HALE (65·7 [62·7–68·5]) for both sexes in 2019. Papua had the lowest life expectancy (65·2 [62·7–67·9]) and HALE (57·3 [54·3–60·1]) for both sexes.

Table S4. Changes in age-standardised summary exposure values for leading risk factors

Supplementary Table 3 shows the age-standardised percentage changes in SEV for the top ten risk factors from 1990 to 2019 for Indonesia and all provinces. At the national level, notable changes included increases to high systolic blood pressure by 21·9% (95% UI 14·6–29·7), high fasting plasma glucose by 83·4% (70·4–97·2), smoking by 15·4% (7·50–23·6), high body-mass index by 266% (178–404), and kidney dysfunction by 20·7% (8·1–38·4). There were some variations by provinces. For example, age-standardised SEV for high systolic blood pressure increased in all provinces except Aceh, Central Kalimantan, Riau Islands, Maluku, and Papua. Smoking, high fasting plasma glucose, high body-mass index, and kidney dysfunction are among those risk factors that increased in all provinces. Notable findings also include increases in high body-mass index over 200% in all provinces except for Maluku. Age-standardised SEV for low birthweight, short gestation, and household air pollution from solid fuels all decreased at both national and subnational levels for Indonesia, except for Yogyakarta.

Table S5. SDI values for Indonesia and 34 provinces between 1990 and 2019

Supplementary Table 4 contains SDI values for Indonesia and all its provinces between 1990 and 2019.

Table S6. Healthcare Access and Quality Index for Indonesian subnational units

Supplementary Table 5 provides HAQ Index for both sexes for Indonesia and its 34 provinces.

Table S7. Burden of lower respiratory infections, 1990–2019

Supplementary Table 6 contains estimates of deaths and DALYs for lower respiratory infections (age-standardised) for both sexes for Indonesia and all provinces, 1990–2019.

Table S8. Under-5 diarrhoeal and lower respiratory illnesses by province

Supplementary Table 7 includes rankings by provinces for diarrhoeal diseases and LRI. This table shows the burden of diarrhoeal diseases and LRI for both sexes in Indonesia at the subnational level.

Section 8: Supplementary figures

Figure S1. Leading causes of disability-adjusted life-years

Supplementary Figure 1 shows the 25 leading Level 4 causes of DALYs in 1990 and 2019 with their percentage change during the period. IHD, intracerebral haemorrhage, and type 2 diabetes were the three leading causes of DALYs in 2019, compared to diarrhoeal diseases, lower respiratory infections, and drug-susceptible tuberculosis in 1990. The age-standardised DALYs rate for IHD increased by 10·0% (95% UI –9·7 to 31·3), and for type 2 diabetes by 52·1% (30·1–75·9). The age-standardised DALYs rate for intracerebral haemorrhage declined by 13·7% (–1·3 to 27·7). The age-standardised DALYs rate for drug-susceptible TB declined by 71·3% (64b·8–76·1), diarrhoeal diseases by 74·6% (67·5–80·3), neonatal preterm birth by 52·5% (37·8–63·9), and lower respiratory infections by 74·8% (68·1–81·0). Notably, age-standardised DALYs rates for measles plummeted.[21] In 1990, measles was the eighth-leading cause of DALYs in Indonesia; by 2019, measles was ranked 148th. The age-standardised DALYs rate for measles declined 96·2% (94·1–97·8).

Figure S2. Attribution of disability-adjusted life-years to risk factors

Supplementary Figure 2 represents the leading causes of all-ages deaths in Indonesia and their percentage changes from 1990 to 2019 for both sexes.

Figure S3. Deaths and disability-adjusted life-years from leading risk factors

Supplementary figure 3 provides the number of deaths and the percentage of DALYs from the 17 leading risk factors in 2019 for both sexes. High systolic blood pressure, tobacco use, dietary risks, high fasting plasma glucose, and high body-mass index were the top five risk factors for attributable deaths. These risk factors were also the leading risk factors for DALYs. The leading risk factor for DALYs in 2019 – high systolic blood pressure – accounted for nearly 500 000 deaths in 2019 alone.

Figure S4. Decomposition of the probability of death by age and province

Supplementary Figures S4A, S4B, and S4C show the decomposition of change in the probability of death from birth to age 20, 20 to 55, and 55 to 90, respectively, at the national and provincial levels. At the national level, we observed a decline of 6·9 in the probability of death from birth to age 20. The probability of death from birth to age 20 declined in all provinces. Steep declines were observed in West Nusa Tenggara (12·7), West Sulawesi (11·6), South Kalimantan (11·3), and Banten (10·2): each province had a greater than 10% decrease in the probability of death. Changing rates of respiratory infections, HIV/AIDS and sexually transmitted diseases, enteric infections, and maternal and neonatal disorders drove decreases in the probability of death in Indonesia between 1990 and 2019 (Figure S4A). We observed a decline of 5·3% in the probability of death for ages 20 to 55. All provinces had a reduction in the probability of death for ages 20 to 55, with the largest percentage changes observed in West Sulawesi (11·4), Central Sulawesi (9·7), and Jambi (9·7). Slower reductions were observed in Yogyakarta (3·2), North Kalimantan (3·4), and Aceh (3·5). Drivers of these trends varied by provinces. For example, diabetes and cardiovascular diseases led to an increase in probability of death in West Nusa Tenggara and South Kalimantan, but HIV/AIDS and sexually transmitted diseases in North Sulawesi (Figure S4B) contributed to a slight increase in probability of death compared to 1990 rates. The changes in probabilities of death for ages 55 to 90 further indicate the country's epidemiological transition. Reductions in infectious diseases led to a decline in the probability of death, while non-communicable diseases led to increases. The largest observed decline in the probability of death for ages 55 to 90 was observed in Bali (Figure S4C) at 8·6; however, the probability of death increased in other provinces, including Papua (0·4), Banten (1·0), Jakarta (1·6), and Aceh (1·9).

Figure S5. Map of Healthcare Access and Quality Index in Indonesia
Supplementary Figure 5 provides a geographical representation of HAQ Index for Indonesian provinces.

Figure S6. Road traffic deaths rates and percentage changes in Indonesia, 1990–2019
Supplementary Figure 6 is a map of Indonesian provinces that represents the rate of road traffic deaths in 2019, and the percentage change of road traffic deaths, 1990–2019.

Figure S7. Age-standardised disability-adjusted life-years estimates for diarrhoea
Supplementary Figure 7 plots age-standardised DALY estimates for diarrhoea for each province and illustrate the changes from 1990 to 2019.

Section 9: Additional insights

Section 9a: The National Tuberculosis Control Program
Indonesia has a high burden of drug-resistant TB and aims to eliminate TB by 2035. Every five years, the National TB Control Program evaluates the achievements, constraints, and challenges of this campaign at the national, provincial, and local levels. At the national level, the programme recommended strengthening leadership, increasing access to services, controlling risk factors, forming partnerships, and building capacity for community independence. Drug-resistant TB very often remains undetected and untreated, leading to worse TB treatment outcomes and increased mortality. Although drugs for TB treatment are free, patients with drug-resistant TB face higher costs compared to other TB patients due to longer treatment periods and charges for health services, transportation costs, accommodation, and nutrition, and suffer lost income. Researchers, policy makers, and health system personnel need to translate knowledge from research projects into changes to TB programme policy or practices. Short communication lines between researchers and implementers ought to be established to ensure that the results and recommendations of the research are properly translated.

Section 9b: Non-communicable diseases in Indonesia
The high burden from type 2 diabetes and its rapid increase is of concern. Diabetes is a particularly expensive disease to treat and manage. As more people in Indonesia lead lifestyles characterised by poor diet and limited physical activity, which correspond to high body-mass index and greater obesity rates, then outcomes such as diabetes and CVD are likely to increase. For example, research shows that the cost of diabetes rapidly increased in Indonesia. The disease is also associated with high disability. Indonesia needs national and subnational plans to stem the tide of diabetes before it becomes a full-blown epidemic. Policymakers need to invest in improving clinical knowledge of diabetes among primary care providers. Early detection is critical. If individuals are aware of their increased risk and people with glucose intolerance are identified earlier, they are more likely to adopt behavioural changes to avoid the disease and its complications. The Indonesian government launched national campaigns against hypertension, diabetes, and obesity. The National Health Social Security Agency (*Badan Penyelenggara Jaminan Sosial Kesehatan, BPJS Kesehatan*) initiated the Chronic Disease Management Program (*Program Pengelolaan Penyakit Kronis*, PROLANIS) in 2010. Until 2020, BPJS Kesehatan registered 224 million people under the universal coverage system. BPJS aimed to screen all registered participants to achieve at least 75% who visit the first-level health facilities will have good results on type 2 diabetes examinations. Participants who have proven hypertension and type 2 diabetes are offered medical

consultations, short message service reminders, home visit, club activities, and diabetes status monitoring.

Section 10: Contributions

Managing the overall research enterprise

Christopher J L Murray, Ali Mokdad, Simon I Hay, Ruri Syailendrawati.

Writing the first draft of the manuscript

Nafsiah Mboi, Ali Mokdad, Samuel Ostroff, Ruri Syailendrawati, Iqbal Elyazar.

Primary responsibility for applying analytical methods to produce estimates

Ali H Mokdad.

Primary responsibility for seeking, cataloguing, extracting, or cleaning data; designing or coding figures and tables

Scott D Glenn.

Providing data or critical feedback on data sources

Qorinah Estiningtyas Sakilah Adnani, Budi Aji, Ernoiz Antriyandarti, Iqbal Elyazar, Arief Hargono, Endang Indriasih, Soewarta Kosen, Dian Kusuma, Santi Martini, Nafsiah Mboi, Ali H Mokdad, Wahyu Nugraheni, Samuel Ostroff, Helena Ullyartha Pangaribuan, Agus Sudaryanto, Indang Trihandini.

Developing methods or computational machinery

Scott D Glenn, Simon Hay, Ali H Mokdad, Christopher J L Murray.

Providing critical feedback on methods or results

Qorinah Estiningtyas Sakilah Adnani, Budi Aji, Ferry Efendi, Iqbal Elyazar, Harapan Harapan, Simon Hay, Dian Kusuma, Agung Dwi Laksono, Santi Martini, Nafsiah Mboi, Ali H Mokdad, Dina Nur Anggraini Ningrum, Wahyu Nugraheni, Samuel Ostroff, Agung Purnomo, Tety Rachmawati, Setyaningrum Rahmawaty, Betty Roosihermiatie, Yoseph Leonardo Samodra, Agus Sudaryanto, Ingan Ukur Tarigan, Jansje Henny Vera Ticoalu, Indang Trihandini.

Drafting the work or revising is critically for important intellectual content

Qorinah Estiningtyas Sakilah Adnani, Rozana Ika Agustiya, Budi Aji, Pungkas Bahjuri Ali, Luna Amalia, Ansariadi Ansariadi, Ernoiz Antriyandarti, Irfan Ardani, Ratih Ariningrum, Ni Ketut Aryastami, Djunaedi Djunaedi, Ferry Efendi, Iqbal RF Elyazar, Nelsensius Klau Fauk, Ghozali Ghozali, Scott D Glenn, Nariyah Handayani, Harapan Harapan, Arief Hargono, Agus DWI Harso, Simon I Hay, Hartanti Dian Ikawati, Endang Indriasih, Karlina Karlina, Soewarta Kosen, Asep Kusnali, Dian Kusuma, Agung Dwi Laksono, Melyana Lumbantoruan, Merry Lusiana, Santi Martini, Nafsiah Mboi, Meilinda meilinda, Ali H Mokdad, Rofingatul Mubasyiroh, Christopher J L Murray, Dede Anwar Musadad, Dina Nur Anggraini Ningrum, Wahyu Pudji Nugraheni, Syarifah Nuraini, Ni'matun Nurlaela, Samuel M Ostroff, Helena Ullyartha Pangaribuan, Indah Pawitaningtyas, Agung Purnomo, Herti Windya Puspasari, Nurul Puspasari, Tety Rachmawati, Setyaningrum Rahmawaty, Hasnani Rangkuti, Betty Roosihermiatie, Tita Rosita, Rustika Rustika, Yoseph Leonardo Samodra, Jenny Veronika Samosir, Siswanto Siswanto, Karen Houston Smith, Agus Sudaryanto, Sugianto Sugianto, Suparmi Suparmi, Ni Ketut Susilarini, Ruri Syailendrawati, Ingan Ukur Tarigan, Jansje Henny Vera Ticoalu, Indang Trihandini, Laksono Trisnantoro, Mugi Wahidin, Tati Suryati Warouw, Retno Widyastuti, Ratna DWI Wulandari.

Managing the estimation or publications process

Simon Hay, Ali H Mokdad, Samuel Ostroff, Ruri Syailendrawati.

Section 11: GATHER table

GATHER Checklist

Item #	Checklist item	Reported on page #
**Objectives and funding**		
1	Define the indicator(s), populations (including age, sex, and geographic entities), and time period(s) for which estimates were made.	8-10
2	List the funding sources for the work.	3
**Data Inputs**		
For all data inputs from multiple sources that are synthesised as part of the study:		
3	Describe how the data were identified and how the data were accessed.	8-10
4	Specify the inclusion and exclusion criteria. Identify all ad-hoc exclusions.	8-10
5	Provide information on all included data sources and their main characteristics. For each data source used, report reference information or contact name/institution, population represented, data collection method, year(s) of data collection, sex and age range, diagnostic criteria or measurement method, and sample size, as relevant.	8-10, appendix
6	Identify and describe any categories of input data that have potentially important biases (e.g., based on characteristics listed in item 5).	18
For data inputs that contribute to the analysis but were not synthesised as part of the study:		
7	Describe and give sources for any other data inputs.	n/a
For all data inputs:		
8	Provide all data inputs in a file format from which data can be efficiently extracted (e.g., a spreadsheet rather than a PDF), including all relevant meta-data listed in item 5. For any data inputs that cannot be shared because of ethical or legal reasons, such as third-party ownership, provide a contact name or the name of the institution that retains the right to the data.	8
**Data analysis**		
9	Provide a conceptual overview of the data analysis method. A diagram may be helpful.	n/a
10	Provide a detailed description of all steps of the analysis, including mathematical formulae. This description should cover, as relevant, data cleaning, data pre-processing, data adjustments and weighting of data sources, and mathematical or statistical model(s).	8-10
11	Describe how candidate models were evaluated and how the final model(s) were selected.	n/a
12	Provide the results of an evaluation of model performance, if done, as well as the results of any relevant sensitivity analysis.	n/a
13	Describe methods for calculating uncertainty of the estimates. State which sources of uncertainty were, and were not, accounted for in the uncertainty analysis.	8-10
14	State how analytic or statistical source code used to generate estimates can be accessed.	Available through GHDx link http://ghdx.healthdata.org/gbd-2019
**Results and Discussion**		
15	Provide published estimates in a file format from which data can be efficiently extracted.	n/a
16	Report a quantitative measure of the uncertainty of the estimates (e.g. uncertainty intervals).	10
17	Interpret results in light of existing evidence. If updating a previous set of estimates, describe the reasons for changes in estimates.	11-14
18	Discuss limitations of the estimates. Include a discussion of any modelling assumptions or data limitations that affect interpretation of the estimates.	18
